# Sunbathing, a possible risk factor of murine typhus infection in Greece

**DOI:** 10.1371/journal.pntd.0009186

**Published:** 2021-03-12

**Authors:** Stavroula Labropoulou, Ekatherina Charvalos, Stylianos Chatzipanagiotou, Anastasios Ioannidis, Panagiotis Sylignakis, Styliani Τaka, Ioulia Karageorgou, Maria Linou, Giota Mpizta, Andreas Mentis, Sophie Edouard, Didier Raoult, Emmanouil Angelakis

**Affiliations:** 1 Laboratory of Medical Microbiology, Hellenic Pasteur Institute, Athens, Greece; 2 IASO Medical Research (IMR) department, IASO Gynecology Obstretrics and Pediatrics Hospital, Marousi, Greece; 3 Department of Biopathology and Clinical Microbiology, Aeginition Hospital, Medical School, National and Kapodistrian University of Athens, Athens, Greece; 4 Department of Nursing, Faculty of Health Sciences University of Peloponnese, Tripoli, Greece; 5 Pananio—Venizelio General Hospital of Heraklion, Heraklion, Greece; 6 Allergy Department, 2nd Pediatric Clinic, National and Kapodistrian University of Athens, Athens, Greece; 7 Aix Marseille Univ, IRD, IHU Méditerranée Infection, MEPHI, Marseille, France; 8 Aix Marseille Université, IRD, APHM, VITROME, IHU-Méditerranée Infection, Marseille, France; Baylor College of Medicine, UNITED STATES

## Abstract

**Background:**

There are few studies about the presence of murine typhus in Greece. Our objective was to conduct a large scale retrospective investigation to determine the clinical and epidemiological features of patients diagnosed with murine typhus in Greece.

**Methodology/Principal findings:**

From 2012 to 2019 serum samples from hospitalized patients and outpatients throughout Greece suspected for murine typhus infection were tested by immunofluorescence assay for *Rickettsia typhi*. Immunofluorescence positive samples obtained since 2016 were also tested by qPCR targeting *R*. *typhi*. Clinical and epidemiological data were retrospectively collected for the patients with confirmed murine typhus. Overall, we tested 5,365 different patients and, in total, 174 patients from all geographic regions of Greece were diagnosed with murine typhus. The most frequently reported sign or symptom was fever (89%), followed by headache (84%) and rash (81%). The classical triad of fever, headache, and rash was present in 72% of patients during their illness. Severe infections with complications including acute renal failure or septic shock were not recorded. The majority of cases (81%) occurred during May–October and peaked in June and September. Most of patients (81%) infected in Athens, recalled that their only activity the last weeks before symptoms onset was swimming on the beach and 59% of them also reported an insect bite while sunbathing.

**Conclusions/Significance:**

Our results may reflect the reemergence of murine typhus in Greece and we highlight the importance of awareness of this difficult-to-recognize undifferentiated febrile illness.

## Author summary

Murine typhus, a febrile disease caused by *Rickettsia typhi*, is often misdiagnosed due to its non-specific presentation. Evidence suggests that murine typhus is more prevalent in Greece than originally believed. We retrospectively collected clinical and epidemiological data for all the patients with confirm and probable murine typhus diagnosed in our laboratory the last eight years and we found evidence of murine typhus throughout Greece. Comparing this evidence to other studies, we found that most of Greek patients presented rash and the typical triad probably due to the awareness of physicians, as during our interrogation, most of physicians mentioned that they considered murine typhus based on rash appearance. Finally, we found that most murine typhus cases in the city of Athens were associated with sunbathing on the beach and we raise the question of beach littering in murine typhus endemic areas.

## Introduction

Murine typhus, an acute zoonotic infection caused by *Rickettsia typhi*, is transmitted to humans by *Xenopsylla cheopis*, a flea that infests rats [1]. The emergence of murine typhus has become apparent mostly in the field of travel medicine, as increasing numbers of individuals are being exposed [2,3]. Indeed, the 2.5% of international travelers reported to the GeoSentinel surveillance network from 1996 to 2008 were from patients with typhus group rickettsioses [4]. The disease occurs worldwide and is underdiagnosed and largely under-reported probably because of its non specifιc clinical manifestations and frequently mild course, a lack of active monitoring and the limited awareness among physicians. The clinical features of murine typhus are non-specific and include fever, rash, headache, myalgia and gastrointestinal symptoms [5,6]. While the clinical course of murine typhus is typically uncomplicated, serious complications have been associated with acute infections, such as central nervous system abnormalities. The death rate is generally low but can reach 4% without the use of antibacterial drugs [6,7].

Murine typhus was first described in Greece in 1932 and, in 1948, 1,420 cases of murine typhus and 17 deaths were reported [8]. Since then, the disease is considered endemic on the Greek islands of Crete [9] and Euboea [10] whereas sporadic cases have been reported in northern Greece [11] and in the island of Kasos [12]. Because of murine typhus nonspecific clinical presentation and the lack of awareness by physicians we raised the question of the under-diagnosis of *R*. *typhi* infections in Greece. As a reference laboratory for the diagnosis of rickettsial infections in Greece, we routinely receive serum samples from patients suspected of having murine typhus. To gain a better understanding of murine typhus in Greece, we retrospectively collected clinical and epidemiological data for all the patients diagnosed with *R*. *typhi* in our laboratory the last eight years.

## Materials

### Ethics statement

This study is based on routine diagnosis samples. All collected data were anonymized in standardized forms according to the Ethic and Scientific Committee of the Hellenic Pasteur Institute under registration number EIP-GDPR-E01.01.

### Patients

We studied serum samples from patients with suspected murine typhus that were sent to our laboratory from January 2012 to December 2019. We received sera from hospitalized patients and outpatients with suspected rickettsioses throughout Greece. For each patient, an acute-phase serum sample was obtained within three weeks after the onset of symptoms and, when possible, a convalescent-phase serum sample (i.e., one collected >2 weeks after onset of symptoms) was also obtained. Clinical data, medical history and complications during illness, habitat and environmental characteristics, presence of pets, livestock, or rodents, daily activities in the 30 days before symptom onset and travel during the previous one month were documented with structured conversations for *R*. *typhi* positive patients by physicians thanks to phone calls.

### *R*. *typhi* antigen preparation

*R*. *typhi* (strain Wilmington) was grown in confluent HEL cell monolayers using 12-mm round coverslips seeded with 1 ml of medium containing 50,000 cells and incubated in a 5% CO2 incubator at 37°C. The density of *R*. *typhi* was assessed by microscopic observation of Gimenez stained smears. The supernatants of 15 flasks were pelleted by centrifugation at 5000g for 15 min and resuspended in 1 mL of phosphate-buffered saline (PBS) at pH 7.3 with 0.1% formaldehyde. Cells were fragmented by sonication, and cellular debris was removed by two successive centrifugations (100g, 10 min each). After the supernatants were centrifuged through 20 mL of PBS with 25% sucrose (6000g, 30 min), the resulting pellet was rinsed three times in PBS (6000g, 10 min) and, using spectrophotometry, was suspended in sterile distilled water at a concentration of 2 mg protein/mL prior to being frozen at − 20°C.

### Serology assays

Patients’ sera at a dilution of 1:50 and 1:100 were firstly screened against *R*. *typhi* by immunofluorescence assay (IFA) using conjugate-anti-Human IgG/IgA/IgM-FITC as previously described [13,14]. Positive and negative controls were diluted at 1:25 and 1:50. Sera found positive against *R*. *typhi* at a 1:50 dilution were further entered into the quantification test and tested for IgG and IgM. For quantification of IgG and IgM, patients’ sera were initially diluted to 1:8 in PBS milk and subsequently diluted in twofold dilutions as previously described [13,14]. Finally, to exclude false-positive results due to cross-reactivity between *R*. *typhi* and spotted fever group (SFG) rickettsioses all murine typhus positive patients diagnosed from 2016 to 2019 were also tested by IFA for SFG *R*. *conorii conorii* antigens as previously described [13,14]. When cross-reactions were observed, a rickettsial antigen was considered to represent the infectious agent if cumulated titres of IgG and IgM antibodies against this antigen were at least twice as high as those of the others. All reactions were performed in dark room and at least two people performed each microscopical examination.

### Molecular assays

Total genomic DNA was retrospectively extracted from samples obtained from 2016 to 2019 with a positive *R*. *typhi* IFA using a QIAamp tissue kit (Qiagen, Hilden, Germany). Samples were handled under sterile conditions to avoid cross-contamination. Samples were screened by qPCR targeting a fragment of the Rpr 274P gene coding for a hypothetical protein, as described previously [15]. Positive results were confirmed by qPCR targeting the glycosyltransferase gene using a previously described Rpr 331 system [15]. A maximum of 10 samples were tested along with negative controls (DNA from IFA negative sera and sterile water) and a positive control (DNA from *R*. *typhi Wilmington* (ATCC VR-144). The quality of DNA handling and extraction of human samples was verified by qPCR for a housekeeping gene encoding beta-actin [10]

### Case definition

A confirmed case of murine typhus was defined as a patient meeting the minimum presumptive clinical criteria and having 1) a positive molecular assay, 2) a single serum with antibody titers of ≥1:64 for IgM and ≥1:128 for IgG antibodies [13,14], acute and convalescent sera showing 3) a seroconversion or 4) a fourfold or greater increase in titers. A probable case of murine typhus was defined as a patient having a single serum with an indirect immunofluorescence assay antibody titer of >1:128 and a clinically compatible illness.

### Statistical analysis

EpiInfo version 6.0 software (Centers for Disease Control and Prevention, Atlanta, GA, USA) was used for significance variations in the number of positive patients between two consecutive months, nonconsecutive months and seasons. Seasons were defined as winter (January–March), spring (April–June), summer (July–September), and autumn (October–December). The Mantel-Haenszel test or the Fisher exact test was used to test for significance. A *p* value <0.05 was considered significant.

## Results

We tested 5,506 different sera obtained from 5,365 patients and we identified 172 patients positive by *IFA* (Table 1). In addition, we realised 133 qPCR and a positive result was obtained for 12 (9%) serum samples. Indeed, 11 acute and 1 convalescent-phase sera were positive by qPCR. For two patients qPCR was positive although serology was negative (Table 2). As a result, in total we diagnosed 174 (98 confirmed and 76 probable) patients with murine typhus. The median age ± interquartile range (IR) of patients was 53 ± 15 years, (range: 1–91 years) and 53% were males.

**Table 1:** *Rickettsia typhi* results of serology and PCR assays.

	Patients tested	Number of realized qPCR (positives)	Serology positive	Total cases
**Acute serum**	5,224	115 (11)	166	168
**Convalescent-phase serum**	141	18 (1)	19	19
**Total patients**	5,365	115 (11)	172	174

**Table 2:** IF assay antibody titers for the 174 confirmed murine typhus patients.

IFA titers	Acute serum (qPCR positive)	Convalescent serum (qPCR positive)	Total patients
IgM *<*1:64 and IgG *<*1:128	2 (2)	0	2
IgM ≥1:64 and IgG *<*1:128	63 (5)	1	63
IgM *<*1:64 and IgG ≥1:128	87 (1)	13	89
IgM ≥1:64 and IgG ≥1:128	16 (3)	5 (1)	20
Total	168	19	174

### Geographical distribution of cases and epidemiological data

**Fig 1:**
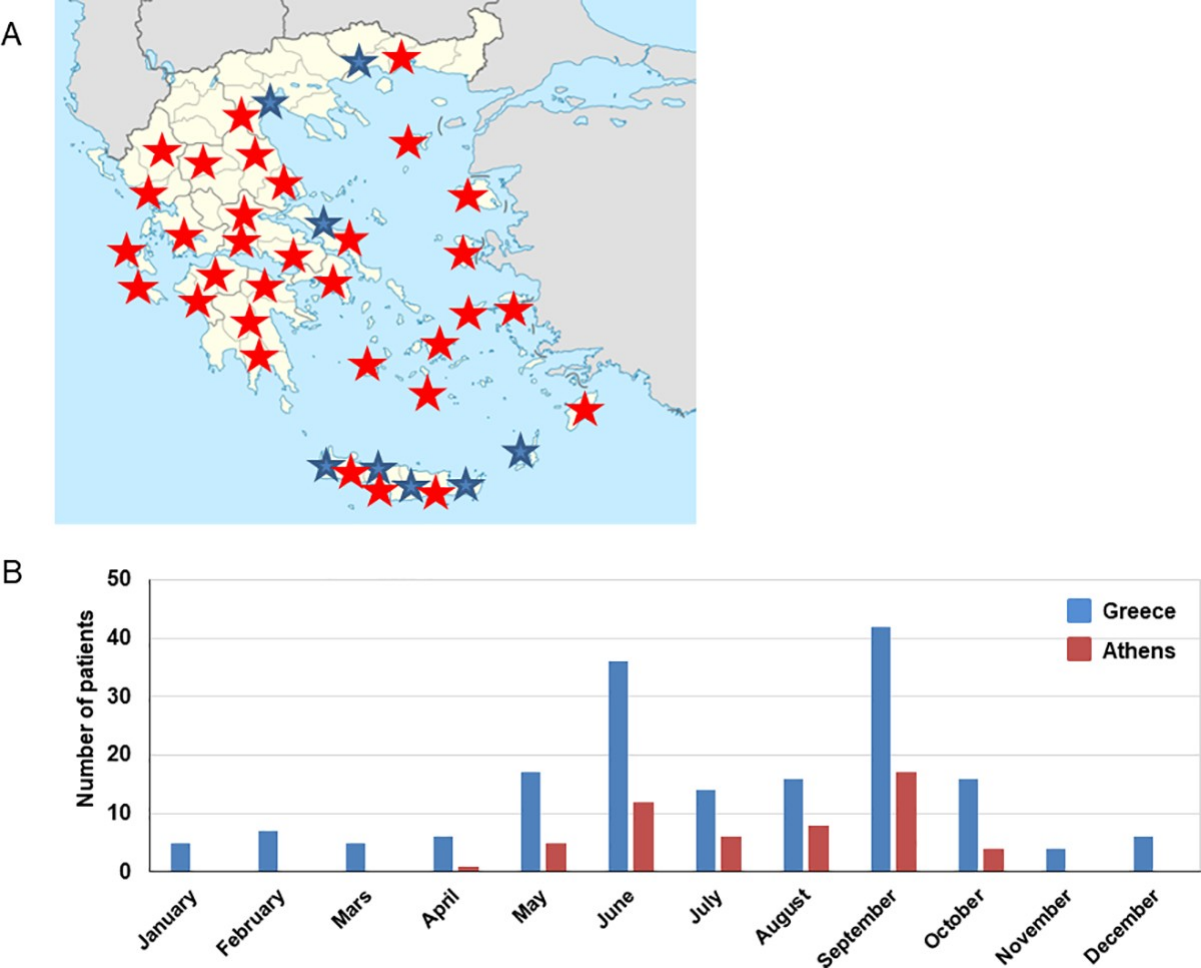
A, Murine typhus patients throughout Greece; B, Seasonal distribution of patients diagnosed for murine typhus, in Greece and in Athens, 2012–2019. Red stars: present murine typhus cases. Blue stars: previous murine typhus reported cases based on the researched of PubMed for peer-reviewed, articles published in English up to June 2020. The search terms were combinations of “murine typhus”, “*Rickettsia typhi*” and “Greece”.

Overall, we found 171 autochthonous murine typhus patients whereas two patients reported a recent travel to Cyprus and one had recently returned from Uruguay. Totally, autochthonous cases were recorded to 33 (58%) out of the 54 prefectures of Greece (Fig 1A). Moreover, we found that murine typhus distribution varied widely among Greek regions and the highest percentage of patients was observed in Central Greece (n = 70, 40%) followed by the region of Peloponnese (n = 29, 17%) (Table 3). Epidemiological information was obtained for 118 patients and among the recorded cases 49 (42%) reported living in rural areas and 19 (16%) were farmers in contact with livestock animals. In total, 101 (86%) mentioned recent/constant outdoor activities, 58 (49%) recalled an exposure to insects or an insect bite, 66 (56%) a close contact with animals and 9 (7%) patients a contact with rodents. Finally, two patients reported a recent travel to Cyprus and one had recently returned from Uruguay.

**Table 3:** Murine typhus patients among the nine greek regions.

Regions	Number of patients (%)
Aegean Islands	16 (9%)
Central Greece	70 (40%)
Crete	16 (9%)
Epirus	14 (8%)
Macedonia	4 (2%)
Ionian Islands	4 (2%)
Peloponnese	29 (17%)
Thessaly	18 (10%)
Thrace	3 (2%)

### Seasonality

Positive murine typhus cases were plotted for each month to identify seasonal distributions from 2012 through 2019 (Fig 1B). Monthly cases of murine typhus cases were lowest from November through April, followed by significant increases during June (*p* = 0.002) and during September (*p* <0.001). During October—November, the number of cases decreased significantly (*p* = 0.005), then plateaued from November through April. Cases increased slightly in April, then increased significantly during May—October (*p* = 0.002). The number of murine typhus cases was significantly higher in summer (July—September) that in autumn (October—December) (*p*<0.0001) and in spring (April—June) than in winter (January—March) (*p*<0.0001). The number of cases did not differ significantly from spring to summer (*p* = 0.07).

### Murine typhus in Athens, Attica region

Totally, 53 (30%) murine typhus patients were diagnosed in the city of Athens. The seasonality of murine typhus cases slightly differed for Athens than for other regions, as all cases were observed from April through October (Fig 1B). Epidemiological information was obtained for 43 patients and eight reported a recent travel in Greece outside Athens whereas three reported a travel abroad. We considered that 32 patients were infected in/or close to Athens and among these only six (19%) reported outdoor activities. Surprisingly, all the rest of these patients (81%) mentioned that their only activity the last weeks before symptoms onset was swimming on the beach and they did not have any other murine typhus associated risk factors. Moreover, 12 (59%) of them also recalled an insect bite while sunbathing. None of these patients mentioned a direct contact with rodents.

### Clinical manifestations

Clinical information was obtained for 61 patients and for 31 (51%) of them a hospitalization was necessary. This high percentage of hospitalization is probably because we obtained clinical data mostly from patients who had previously presented to hospitals. The most frequently reported sign or symptom was fever (89%), followed by headache (84%) and rash (81%) (Table 4). The duration of fever ranged from 4 to 15 days (38 to 40°C) whereas the rash was macular or maculopapular with a duration from three to 10 days. The classical triad of fever, headache, and rash was present in 72% of patients during their illness. Other less common manifestations were cough (67%), anorexia (45%) and arthralgia (35%). Gastrointestinal tract symptoms like nausea or vomiting was found in 38% of cases, and diarrhea in 26% of cases. Neurological manifestations were also reported, including confusion in 7% and photophobia in 4%. Liver or spleen enlargement was observed in 4% of patients. All hospitalized patients presented pulmonary manifestations including pneumonia or pulmonary infiltrates. Finally, severe infections with complications including acute renal failure or septic shock were not recorded.

**Table 4:** Clinical characteristics of murine typhus patients.

Clinical signs and symptoms	Number of patients (%)
Fever	54 (89%)
Headache	51 (84%)
Rash	49 (81%)
The classic triad*	44 (72%)
Cough	41 (67%)
Pneumonia or pulmonary infiltrates	37 (61%)
Anorexia	27 (45%)
Nausea/vomiting	23 (38%)
Myalgia/arthralgia	21 (35%)
Diarrhea	17 (26%)
Confusion	4 (7%)
Photophobia	2 (4%)
Hepatosplenomegaly	2 (4%)

## Discussion

We report a large series of *R*. *typhi* infections and we found evidence of murine typhus throughout Greece. Our immunofluorescence and molecular assays were sensitive and versatile and have been routinely used for the detection and diagnosis of murine typhus [13–15]. Furthermore, we routinely included large numbers of negative controls in our assays that were processed identically to the test samples. Serological tests are the easiest methods for the diagnosis of *R*. *typhi* infection but seroconversion is usually detected 7–15 days after disease onset [2]. We detected two samples positive by qPCR but negative by IFA and a limitation of our study was that samples were retrospectively tested using molecular assays as we have managed to test only immunofluorescence positive samples since 2016. Indeed, it is very possible that this number could be higher if sera were routinely tested by qPCR on the basis of both clinical and epidemiology data also. Finally, we cannot exclude that some patients were false-positive diagnosed with murine typhus before 2016 as only samples after 2016 were also tested for SFG rickettsioses.

Previously in Greece, *R*. *typhi* infections were considered endemic in some Greek regions [9,10] whereas for Europe, exposure to *R*. *typhi* has been recorded in Spain, Canary Islands [16], Cyprus [17], Italy [18], and Croatia [19]. Although autochthonous cases of murine typhus have not been described in the countries of North Europe, sporadic cases are commonly identified in travelers who visited endemic areas [7,15,20]. In Cyprus although to date many cases of murine typhus have been described, the first confirmed case was reported in a Swedish woman who developed fever, severe headache, myalgia and rash, three weeks after a visit to Cyprus [21].

The diagnosis of murine typhus has been characterized as a challenge because many physicians are unfamiliar with the nonspecific symptoms during the early stages of illness. Rash is considered to be the most typical finding of murine typhus but its prevalence varies from 28–82% depending on the geographic region. Indeed, rash was found to 20% of patients from Thailand, 38% of patients from Laos, 49% from Texas, 63% from Spain and 80% from Greece [2,6]. Similarly, in previous studies the typical triad for murine typhus occurred in less than half of the patients [6,7,22]. In our study, most of patients presented rash and the typical triad. We believe that this higher rate of rash as a symptom is due to the awareness of physicians, as during our interrogation, most of physicians mentioned that they considered murine typhus based on rash appearance.

Our findings show that the number of murine cases in Greece varies by season. In studies where seasonal variation was recorded, at least half of cases were diagnosed during summer and early autumn [6,15]. A similar seasonality was also previously described in Greece where most cases occurred during summer with a peak on the late summer [10]. In other studies, the highest incidence was observed in May and June [23,24] suggesting that climatic conditions are the main factors contributing to different seasonal distributions of human cases in different geographical areas. Human infection with murine typhus has been associated with the presence of rats and their fleas. This probably explains the two seasonal peaks during June and September that we observed due to the higher abundance of *X*. *cheopis* in our country during these months possibly because rats are reproducing these periods.

**Fig 2:**
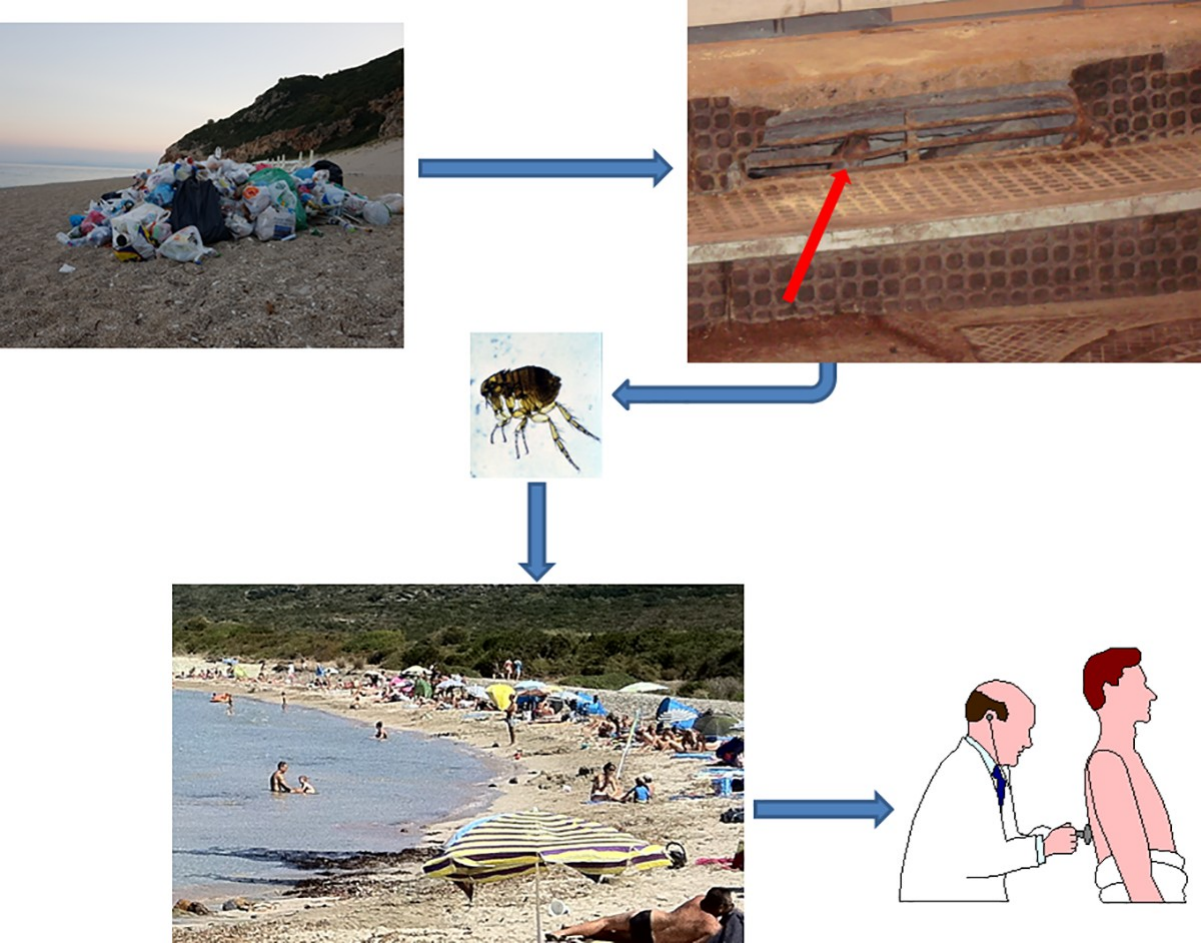
Sunbathing on the beach and murine typhus.

Surprisingly we observed that most cases of murine typhus in Athens were associated with sunbathing on the beach. As the most of the reported human cases of murine typhus have been associated in sites with large rat populations we believe that during summer rats infest beach environments probably because of littering and humans are infected by the rat fleas while sunbathing (Fig 2). Although adult *X*. *cheopis* spend most of their time on their host, they can survive for relative long time in off-host environments [25]. Indeed unfed adult *X*. *cheopis* survived for 38 days after emergence while those given blood meals lived for 100 days [26]. Survival of *X*. *cheopis* depends also on climatic conditions and their population decreased quickly during hot, dry weather [25].

In conclusion, we provide evidence that *R*. *typhi* is widespread in Greece. Our data affect local practice as we found that physicians consider murine typhus mostly when rash is presented. Murine typhus cases are frequently described to travelers visiting Greece [4,7] and although further work needs to be done to confirm our hypothesis, we believe that some of the travelers are possibly infected on the beach environments considering that tourists spend most of their time on the beach side. Finally, we raise the question of beach littering in murine typhus endemic areas as it is associated with increased rodent populations and as a consequence increased risk of infection by *R*. *typhi*.
